# 

**Published:** 1801-11

**Authors:** 


### To CORRESPONDENTS.

*Dr.* Scott *will perceive that his reference is given by Dr.* Fletcher, *in p.* 312 *of our laft Number.*

*Mr.* Syer'*s Cases, and Mr.* Reece'*s Communication, (with an Engraving of his Tooth-Instrument) shall appear in our next.*

---

### ERRATA.

Page 374. l. 16, *for* by ammonia, *read* by carbonat of ammonia.
l. 20, *for* foluble, *read* infoluble.

---

### ADVERTISEMENT.

*Persons who reside abroad, and who wish to be supplied with this Work every month as published, may have it sent to them,* FREE OF POSTAGE, *to New York, Halifax, Quebec, and every part of the West Indies, at three guineas per annum, by Mr.* THORNHILL, *of the General Post Office, at No.* 21, *Sherborne Lane; to Hamburgh, Lisbon, Gibraltar, and every part of the Mediterranean, at three guineas per annum, by Mr.* BISHOP, *of the General Post Office, at No.* 22, *Sherborne Lane; to the Cape of Good Hope, or to any part of the East Indies, at two guineas per annum, by Mr.* GUY, *of the India House; and to any part of Ireland, at two pounds ten shillings per annum, by Mr.* SMITH, *of the General Post Office, at No.* 3, *in Sherborne Lane. It may also be had of all persons who deal in Books in every part of the world.*

